# Organ-on-chip applications in drug discovery: an end user perspective

**DOI:** 10.1042/BST20210840

**Published:** 2021-08-16

**Authors:** Naomi Clapp, Augustin Amour, Wendy C. Rowan, Pelin L. Candarlioglu

**Affiliations:** 1 Refractory Respiratory Inflammation Discovery Performance Unit, GlaxoSmithKline, Gunnels Wood Road, Stevenage, Hertfordshire SG1 2NY, U.K.; 2 Novel Human Genetics Research Unit GlaxoSmithKline, Gunnels Wood Road, Stevenage, Hertfordshire SG1 2NY, U.K.; 3 In vitro/In vivo Translation-Complex In Vitro Models, GlaxoSmithKline, Gunnels Wood Road, Stevenage, Hertfordshire SG1 2NY, U.K.

**Keywords:** drug development, drug discovery, microfluidic models, organ-on-a-chip

## Abstract

Organ-on-chip (OoC) systems are *in vitro* microfluidic models that mimic the microstructures, functions and physiochemical environments of whole living organs more accurately than two-dimensional models. While still in their infancy, OoCs are expected to bring ground-breaking benefits to a myriad of applications, enabling more human-relevant candidate drug efficacy and toxicity studies, and providing greater insights into mechanisms of human disease. Here, we explore a selection of applications of OoC systems. The future directions and scope of implementing OoCs across the drug discovery process are also discussed.

## Introduction

Attrition rates have long been considered as the main cause of costs in drug development, which has been estimated to approximate $1 billion per new medicine entity between 2009 and 2018 [1]. Roughly half of the clinical trial terminations are attributed to a lack of efficacy and a further quarter are related to safety concerns. The lack of clinical translation of preclinical models used for assessing drug efficacy or toxicity is one of the major causes behind the high attrition rates [2]. Clearly, there is an unmet need to update the current preclinical testing paradigm.

**Figure 1.**
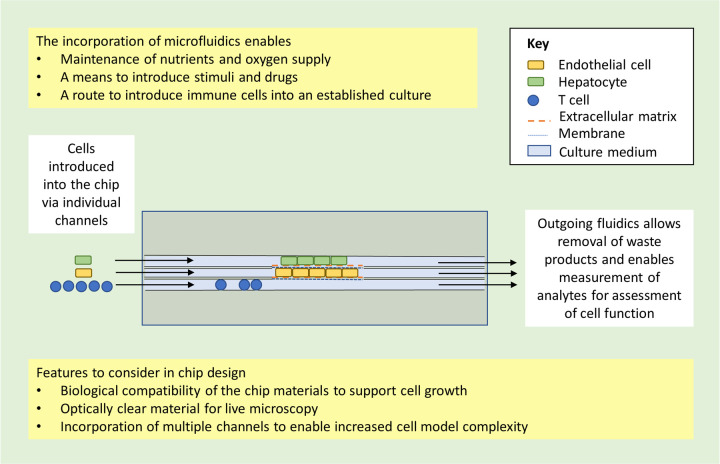
Schematic diagram depicting an example of a basic liver-on-chip model as a representative organ-on-chip (OoC). The active flow within the channel of a chip enables cells to be perfused with media containing oxygen and nutrients (inlet) and the removal of waste products and the sampling of metabolites for assessment of cell function (outlet). This schematic is for illustrative purposes only and does not represent any particular existing OoC model or all possible types of OoC models. It is intended to exemplify some of the basic bioengineering principles required for the development of an OoC unit.

**Figure 2.**
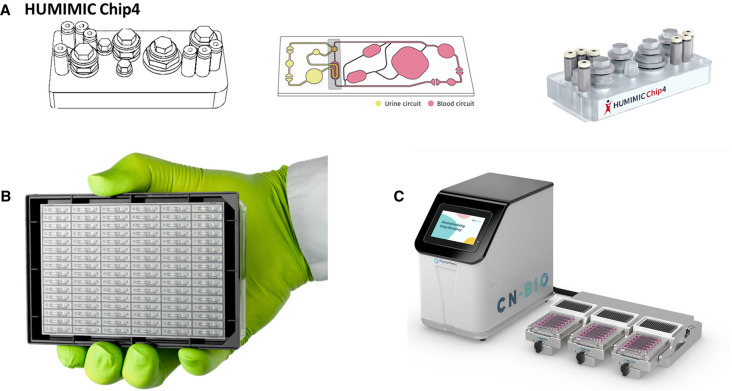
Examples of commercialised organ-on-chip (OoC) systems. (**A**) The HUMINIC chip 4 manufactured by Tissuse. (**B**) The OrganoPlate® 2-lane 96 manufactured by MIMETAS. (**C**) The PhysioMimix™ OoC manufactured by CN-Bio. The pictures are reprinted courtesy of the manufacturers and with their permission.

Motivation to address these limitations has given rise to the organ-on-chip (OoC) technology: microfluidic, chip-based, three-dimensional (3D) cell culture models with an active flow (Figure 1). In recent years, commercially available OoCs have been increasingly integrated within the drug development phase, to replace more traditional preclinical models (Figure 2; [3–6]).

**Figure 3.**
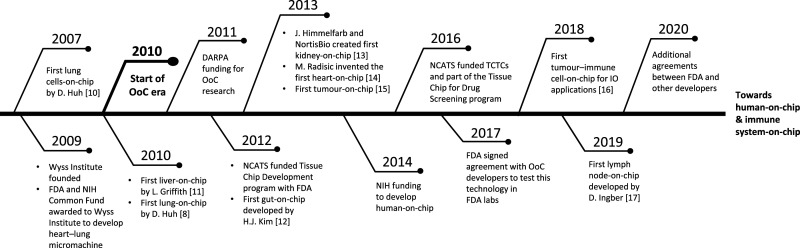
Timeline for the evolution of organ-on-chip (OoC). The first OoC model to accomplish organ-level functionality, tissue–tissue interactions and a physiologically relevant organ microenvironment with vascular perfusion came in 2007 as a lung-on-chip by Huh et al. Since then, funding from organisations such as DARPA and NIH, and the founding of Wyss Institute, has propelled OoC technology from a nascent idea to a rapidly growing area of research with potential for utility across the drug discovery process. DARPA, Defence Advanced Research Projects Agency; FDA, US Food and Drug Administration; IO, immuno-oncology; MEM, micro electromechanical system; microTAS; miniaturised total chemical analysis system; NIH, National Institute of Health; PDMS, polydimethylsiloxane; TCTCs, Tissue Chip Testing Centers.

OoCs originated from the development of miniature devices compatible with cell culture and imaging [7] with the earliest OoC developed in 2010 by Donald Ingber's group [8]. The concept evolved from a research interest in the mechanical control of tissue and organ development [9] and led to the development of a lung-on-chip device; this integrated different tissues on the same chip to replicate the alveolar–capillary interface and recreated a functional, structural and mechanical representation of a lung alveolus. The temporal evolution of OoCs from precursor devices to the current state-of-the-art is depicted in Figure 3 [8,10–17].

These models were initially referred to as microphysiological systems (MPS) and the denomination has since evolved with the technology itself. However, the aim has remained constant: creating tissue or organ functionality, beyond the capabilities of standard 2D or static 3D cell culture systems [18]. While ‘MPS’ and ‘OoC’ are often used interchangeably, the FDA define OoCs as part of a sub-category of MPS; they classify an MPS as an *in vitro* system, with cells isolated from tissues/organs or from organoids, that aims to recreate the physiological microenvironment. In contrast, an OoC is defined as a miniaturised MPS that is ‘engineered to yield and/or analyse functional tissue units capable of modelling specified/targeted organ-level responses’ [19], which is similar to the ORCHID definition [20]. Examples of OoC models replicating the microstructure, mechanical properties and functionalities of living organs have been reviewed [18,21]. Within the chip, one or more cell types are usually cultured within a 3D scaffold where cells can attach to an extracellular matrix (ECM) or porous membranes. The cells are continuously perfused with media containing oxygen and nutrients to maintain their function and survival, and the application of physical cues, such as shear stress, is possible. More recent advances, have enabled structures with higher aspect ratios and sensors, to monitor cellular and environmental changes [22,23].

The initial focus of OoCs was on single organs relevant to toxicology studies such as the kidney [13,24], liver [25,26], lung [8] or heart [27,28] It is now widely accepted that more accurate toxicology readouts may be achieved by incorporating multiple organs, to enable their interaction and more accurate evaluation of downstream metabolites. Efforts to integrate such models to form organ combinations are emerging, with the eventual goal of creating a ‘body-on-chip’ [29–31]. This provides greater scope and offers hope of gaining insight into human disease mechanisms, predicting the safety and efficacy of novel therapies and reducing the use of laboratory animals.

This review discusses the current applications of OoC technology and its scope for implementation in the drug discovery process, as well as its future directions from a drug discovery focussed end user perspective.

## Replicating *in vivo* conditions

The primary aim of developing any OoC model for drug discovery purposes is to replicate *in vivo* physiology and provide a translational *in vitro* model. Despite efforts, to attain true clinical translatability remains a challenge and the technology is limited to a few examples.

One path to overcoming the translational hurdle is through the inclusion in microfluidic systems of tissue-specific environmental cues, such as flow and mechanical stress, recreating the tissue microenvironment in which cells would normally reside *in vivo*. Karalis and co-workers [32] recently showed how cyclic stretch could lead to the development of a gut-on-chip model that is physiologically more relevant than the corresponding static system; the transcriptomic profile of this model more closely resembled that of a gut tissue biopsy than the corresponding non-chip-based organoid.

Maintaining long-term cell viability and functionality *in vitro* has been a major challenge, but continuous perfusion of oxygen and nutrients, and removal of metabolic waste products in OoC models, enable longer-term culture and relevant cell analysis [33,34]. The use of primary cells obtained from multiple donors can capture donor-to-donor variation and provide an important insight into how *in vitro* results may translate to the clinic. In a liver-on-chip model, inter donor variability in metabolic clearance was predicted with albumin, urea, lactate dehydrogenase and cytochrome P450 mRNA levels. The correlation of predicted clearance with *in vivo* values enabled the development of an *in silico* drug metabolism model predicting pharmacokinetic variability [35]. The study provided insight into donor variability; however, the donor number was low and the model could not accurately reproduce the variability of some of the drug clearance parameters measured. It is likely that such models will be refined with the advent of higher throughput OoC models that enable testing of a greater number of donors tested at statistical powers equivalent to clinical trial.

The use of primary cells, where possible, is an important consideration when replicating *in vivo* conditions in OoC models. Obtaining good quality primary cells from tissues such as the central nervous system or a lung alveolus, remains a challenge. Further complexity arises when multiple cell types from a single donor are required to reconstitute organ functionalities. For example, a liver-on-chip model may require combinations of cell types including stellate cells, Kupffer cells, hepatocytes and liver sinusoidal endothelial cells. Induced pluripotent stem cells (iPSCs) are considered as an alternative to primary cells for autologous systems due to their wider availability and amenability to genetic modification [36,37]. Their ability to differentiate into different cell types, retaining single donor characteristics once differentiated, could enable personalised medicine development. There are disadvantages of iPSC technology: dependent on the cell type, the differentiation protocols can be complex and lengthy; iPSC derived cells are often structurally and functionally immature, as exemplified by iPSC derived cardiomyocytes that resemble foetal cardiomyocytes [38]. The development of more refined iPSC differentiation protocols leading to mature cell phenotypes would facilitate the establishment of autologous OoC models.

## Evaluating drug safety and efficacy

One of the main potential applications of OoCs is to assess the safety of drugs prior to entering clinical trials. Only 48% of adverse drug reactions in humans are predicted in preclinical testing [39]. This is in part due to the limited ability of commonly used preclinical species to capture human drug toxicities, as shown for the liver-induced toxicity of diclofenac [40].

A standard approach for the validation of a novel safety model, is to determine the sensitivity and specificity of the model with a set of compounds that have a large body of preclinical and clinical data available [41,42]. Once the dynamic range of the model is established, clinical safety outcomes are then replicated in an acute or chronic state. An immunocompetent liver-on-chip model described by Sarkar et al. [40] incorporated hepatocytes and nonparenchymal cells such as Kupffer cells to assess the acute form of drug-induced liver injury resulting from diclofenac secondary metabolite formation. As these cells typically lack functional longevity in standard static cultures, controlled perfusion via a microfluidic pump and a 3D scaffold, enabled the formation of tissue-like structures, which are critical for maintaining functionality [43]. In the model, diclofenac produced a metabolism and toxicity profile comparable to that observed in humans, but not in animal models. This liver-on-chip model subsequently enabled the development of a viral infection model by virtue of maintaining 40-day functional stability of the system for chronic safety testing [44].

The anti-proliferative effects of cancer drugs on haematopoietic stem cells are also not well-predicted in preclinical animal models. Bone marrow-on-chip models are being developed to examine potential toxicity screening of various compounds, including small molecules and large biologics. The bone marrow is a highly specialised niche composed of an array of cell types with complex phenotypes and these models must mimic the *in vivo* microenvironment and enable the growth of mesenchymal stem cells [45]. In collaboration with AstraZeneca, researchers from the Wyss Institute developed a vascularised bone marrow-on-chip model that successfully captured the dynamic physiology of a healthy bone marrow; the model supported the differentiation of multiple hematopoietic cell lineages over the course of 4 weeks, which reproduced the clinically observed toxicity profile of the inhibitor of aurora B kinase AZD2811. This enabled AstraZeneca to investigate better-tolerated dosing regimens for this compound in the clinic [46]. This model appears promising for evaluating long-term bone marrow toxicities of new chemotherapies and drug combinations. Furthermore, Cohen et al. [47] were able to study the mechanism of the drug-induced nephrotoxic side effects of cisplatin and cyclosporine with a novel kidney-on-chip model. The authors hypothesised that the clinical adverse effects of the two drugs were linked to glucose accumulation. This was validated by retrospectively analysing the clinical data of 247 patients that received cyclosporine or cisplatin in combination with the glucose reabsorption inhibitor empagliflozin, which was found to significantly reduce the incidence of kidney damage when compared with control groups. This work provides a perfect example of the potential for unravelling drug-induced toxicities with OoCs.

Predicting drug efficacy in preclinical models is another major challenge in drug discovery because current animal models do not always replicate well the pathophysiology of human disease. Recreating disease pathology affecting the pulmonary vasculature, for example, is highly challenging. A lung-on-chip model that successfully recreated a functional alveolar–capillary interface paved the way for various applications in the respiratory disease area [8]. The architecture of the lung in idiopathic pulmonary fibrosis, with its patchy stiff fibrotic tissue, has also been modelled by a lung-on-chip model that enables the study of anti-fibrotic drugs in greater detail than possible *in vivo*, such as the inhibitory effect of nintedanib on neo-vascularisation [48].

There has been significant interest in building human *in vitro* OoC models of disease in cases where animal models do not exist. One such example is Hepatitis B virus (HBV) infection; associated with liver cirrhosis and hepatocellular carcinoma, it is a global health concern, with over 240 million people infected worldwide [49]. Our understanding of the host–pathogen interactions is limited by the complexity of establishing a relevant model: patient-derived isolates of primary human hepatocytes (PHH) must be permissive to HBV infection and infection throughout all stages of the viral life cycle must be maintained. To overcome this hurdle, a liver-on-chip was developed to mimic HBV infection [44]. PHH cells were seeded onto the platform; continuous circulation of nutrients and oxygenated media led to the formation of hepatocyte microtissues which could be maintained in culture for at least 40 days, enabling the completion of the viral life cycle. Importantly, HBV infection resulted in innate immune responses, which replicated the clinical outcome in infected patients and supported the use of clinically relevant low viral titres. This aspect demonstrates the potential of OoC models to enable the investigation of immune evasion pathways of viruses, the modelling of drug treatment and the identification of novel clinical biomarkers.

*In vitro* human disease models are now proving suitable for efficacy testing and improving our understanding of molecular mechanisms of disease. Non-alcoholic steatohepatitis (NASH), the most severe form of non-alcoholic fatty liver disease (NAFLD), is a prime example. With the increasing prevalence of diabetes and obesity, NAFLD has become the most common chronic liver disease in developed countries with no specific pharmacological therapeutic options [50,51]. Previously, the mechanism for the association of a genetic variant of the lipase *PNPLA3* in NAFLD was not well understood [52]. PHH and Kupffer cells were cultured on chip with wild-type or *PNPLA3* I148M mutant hepatic stellate cells in the presence of free fatty acids to induce a NASH-like phenotype. In the model, hepatic stellate cells carrying the mutation, potentiated the disease state. The addition of the anti-NASH compound obeticholic acid reduced inflammatory mediators, as observed in clinical trials [53,54]. Those *in vitro* observations from the liver-on-chip model would have not been possible in a static 3D culture model, such as a PHH spheroid model, for several reasons: static models are incompatible with maintaining the physiological function of hepatocytes for prolonged periods and adding Kupffer and stellate cells to hepatocytes without the disruption of the spheroid structure is extremely challenging.

In conclusion, whilst OoCs appear to have tremendous potential for the assessment of drug safety and efficacy, systematic studies comparing the predictive power of available OoCs to those of current methodologies are lacking. It is, therefore, too early to provide a definitive view on the translational relevance of the OoC technology and how it compares to the current approaches in drug discovery.

## Incorporating immune cells

The immune system is influential in the progression of many diseases including cancer, neurodegenerative diseases, chronic infections and autoimmunity. Considering the substantial differences between the human and animal immune system, the ability to incorporate immune cells into OoC systems to model human-specific immune responses to treatments targeting the immune system, such as biologics or cell therapies, will enable immunotoxicity assessments that are otherwise missed in *in vivo* models. The challenge is recapitulating the structural and functional complexity of the human immune system in a relevant manner [55].

The addition of immune cells to tumour-on-chip models enabled the study of the migratory phenotype of activated natural killer cells and their ability to penetrate into a glioblastoma tumour using time-lapse microscopy [56]. In another example, an immune-competent tumour-on-chip model, was used to track interactions between tumour fragments and autologous tumour infiltrating lymphocytes (TILs). Using automated quantitative image analysis, the fraction of cell death attributable to TILs was found to respond to the immune checkpoint inhibitor anti-PD-1 [16]. A hepatocellular carcinoma-on-chip model was used to evaluate the impact of tumour microenvironment conditions on the cytotoxicity of different T cells engineered to express a tumour-specific T cell receptor (TCR). Using this tumour-on-chip, it was possible to detect how T cell-mediated cytotoxicity was influenced by the tumour relevant hypoxic and inflammatory conditions. Optimal T cell-mediated cytotoxicity was observed under normoxic and inflammatory conditions, whereas hypoxia reduced their functionality. This difference in avidity of TCR–T cells was beyond the sensitivity of the 2D well plate-based assay that was run in parallel, showing the potential of OoC in immuno-oncology applications. A vascularised breast cancer-on-chip also enabled the identification of the immunomodulatory effect of cancer-associated fibroblasts on the anti-HER2 antibody, trastuzumab, mediated cytotoxicity [57].

Emulating the immune system in a standalone tissue-on-chip, for example a lymph node-on-chip, would further advance this technology. A simple design has recently been developed, where T cells and dendritic cells can interact with each other in a single channel, on the same plane. This interaction aimed to recapitulate the T cell and antigen-presenting cell cross-talk in the lymph node, which marks the initiation of the adaptive immune response through the appropriate antigen interrogation [58]. Ingber and co-workers [17] further developed a model and used it to evaluate a vaccine response; cell types self-organised into lymphoid follicles, the building blocks of germinal centres where B cells differentiate into antibody-secreting plasma cells. While these studies are promising, the qualification of these lymph node-on-chip models in other settings is important, such as testing the ability to generate antigen-specific neutralising antibodies during pathogen infections.

## Materials and scaffolding

Polydimethylsiloxane (PDMS) is used extensively to manufacture chips and membranes and is advantageous for several reasons [59]. Its gas permeability enables oxygen supply to cells in microchannels, which is particularly beneficial for cultures of primary cells with high metabolic demands such as hepatocytes [60]. Its flexibility enables dynamic forces to be applied to cells: the mechanical stress of respiratory movements can be replicated in lung-on-chip models, where cyclic strain is applied to a PDMS membrane to act as a micro-diaphragm, replicating *in vivo* conditions [32,61]. The optical clarity of PDMS facilitates on-chip immunohistochemical staining and imaging, enabling easy characterisation of microtissues. Despite its versatility, the material properties of PDMS also present challenges. Its high hydrophobicity precludes cell adhesion and chemical or biological modifications are necessary to enable cells adhering to its surface [62,63]. Of importance for pharmaceutical applications, the hydrophobic surface also encourages the non-specific, unpredictable binding of small molecules to its surface, which reduces free drug concentration [64,65]. Another challenge with the use of PDMS in microfluidic systems is the leaching of remaining uncured oligomers into the culture medium and cells which can interfere with biological processes and lead to spurious experimental outcomes [66–68] The field has been looking into alternative materials that are non-absorbent, gas permeable, biocompatible, optically clear and amenable to mass manufacturing. Thermoplastics, hydrogels, glass and biocompatible materials with a long history of being used for tissue engineering, such as polylactic acid (PLA), as well as the combinations of these materials, are investigated to support the next generation of microfluidic chip manufacturing [69–71].

## Increasing general uptake

For a wider adoption of OoCs by the pharmaceutical industry, increased throughput and integration with existing laboratory equipment need to be addressed [72]. In lead optimisation, there is an increasing desire to evaluate potency in more complex models. This is in contrast with the late preclinical phase of clinical candidate identification where numbers of compounds tested are typically in the single-digit range and where *in vivo* relevance becomes a greater priority. The lead optimisation phase is where OoCs could gain traction, if throughput and cost could be improved. The OrganoPlate® platform with a microtitre plate footprint and 96-well capabilities exemplifies more recent trends fulfilling these conditions [73]. Its thin glass-bottom facilitates microscopic imaging and the microtitre plate format is compatible with automated readers and robot handling. Further development of OoC systems with the standard footprint of 24-, 96- or 384-well microtitre plates would ensure their seamless integration within the current robotic solutions and automation equipment already in place in the pharmaceutical research and development environment.

Achieving regulatory acceptance is also key to the mainstream adoption of the technology by end users. Currently, the use of OoC models is limited to preclinical drug discovery, with the data restricted to internal study reports. Given the potential power of OoC platforms in their clinical predictability, it is regrettable that those data are not included in regulatory documents as part of investigational new drug (IND) submission to regulatory bodies. To gain regulatory acceptance, models should have a defined test methodology, proven relevance, qualification and evidence of reliability in their context of use. A list of reference compounds with known toxicology and metabolism data has been compiled by the industry, to aid the qualification of liver-on-chip models [41]. A dialogue is encouraged by regulatory authorities both in Europe and the United States (US); by the European Medicines Agency Innovation Task Force and are also by the US Food and Drug Administration via the Center for Drug Evaluation and Research and the Alternative Methods Working Group. There are also ongoing efforts to qualify OoCs through the National Institute of Health's National Center for Advancing Translational Sciences. Ultimately, these efforts must be replicated globally to accelerate the wider acceptance of OoCs.

## Multi-organ combination *in vitro*

Although standalone OoC models can improve the predictability of preclinical testing, their full potential will be unleashed through multi-organ combinations. Single-organ models are not always fully predictive of the pharmacokinetic and safety properties of drugs, a multi-organ model has enormous potential in reducing or replacing animal usage. One of the first liver-on-chip designs developed at the Massachusetts Institute of Technology by Linda Griffith, has been adopted to create 4-, 7- and 10-way organ models that have demonstrated organ functionality for up to 4 weeks [74]. A similar approach with a 4-organ chip that connects the gut, liver, kidney and bone marrow was used to predict the pharmacokinetic properties of cisplatin [75]. Maschmeyer et al. [76] developed both a 2-organ and 4-organ chip, and used the 4-organ chip to interconnect models of human intestine, liver, brain and kidney derived from iPSCs, using a universal medium to enable the maintenance of the different cell types used for 2 weeks. Multiple examples of interconnected OoC models quickly followed, with an integrated gut–liver [77], female reproductive tract [78] and an 8-OoC combination coupled to an automated culture and sampling system [79]. Although these initial multi-organ models represent promising proof of concept studies, their day-to-day relevance to drug discovery remains to be confirmed. More targeted approaches with 2- to 4-organ combinations enabling specific pharmacokinetic/pharmacodynamic (PK/PD) or absorption, distribution, metabolism and excretion (ADME) modelling are more likely to be initially adopted.

The main bottleneck to developing multi-organ chips lies in chip design and tissue maturation. A potential barrier to interconnecting multiple OoC models is the wide variety of platforms being designed, pointing out a clear need for standardisation. For maximum usability, each module should have connector valves and similar scaling to accurately mimic *in vivo* vascular blood supply and flow rate. The tissue culture media must also be compatible with cell types across all connected models; a challenge, considering the specific requirements of cell types to specialised culture media [45]. Advances in the development of common media for multiple organ-derived cell types have been reported, but these only support short-term co-cultures [30]. Modular ‘plug and play’ systems are being developed to provide flexible connections without leakage. For example, the μOrgano system is a Lego®-like plug and play system which enables the initial culturing of independent OoC systems and subsequent connection to create an integrated multi-organ system [80]. The flexibility to integrate models at different timepoints, opens the opportunity for tissue-specific maturation processes and truly multi-organ chip models. There are multiple engineering challenges, such as different chip design requirements per organ, integrating a vascular connection to each organ and avoiding the introduction of air bubbles or infection risks at connection points.

## Conclusions

OoC technology is still in its early stages of uptake in drug discovery, however, its potential to predict drug safety and efficacy could have significant impact throughout the drug discovery process. The potential of OoC models, to recapitulate the physiological, mechanical and biochemical complexity of human tissues may enable the assessment of in-depth functional parameters beyond the scope of current *in vitro* cultures. The qualification and regulatory acceptance of these models will help to derisk their uptake by the pharmaceutical industry and should facilitate wider adoption. Plug and play immune-competent multi-organ systems manufactured in the right materials, compatible with existing laboratory equipment and meeting the throughput needs of the industry is key for their overall success. Ultimately, adopting a collaborative approach, with academia and industry working in close partnership will be required in order to meet those objectives. New disruptive drug discovery paradigms are on the horizon.

## Abbreviations

HBV: hepatitis B virus
iPSCs: induced pluripotent stem cells
MPS: microphysiological systems
NAFLD: non-alcoholic fatty liver disease
NASH: non-alcoholic steatohepatitis
PDMS: polydimethylsiloxane
PHH: primary human hepatocytes
TCR: T cell receptor
TILs: tumour infiltrating lymphocytes
